# High level of multidrug-resistant *Escherichia coli* in young dairy calves in southern Vietnam

**DOI:** 10.1007/s11250-019-01820-6

**Published:** 2019-02-08

**Authors:** Bui Phan Thu Hang, Ewa Wredle, Stefan Börjesson, Kerstin Svennersten Sjaunja, Johan Dicksved, Anna Duse

**Affiliations:** 1 Department of Animal Sciences and Veterinary Medicine, An Giang University, Long Xuyên, An Giang Province Vietnam; 2 Department of Animal Nutrition and Management, Swedish University of Agricultural Sciences, SE-75007 Uppsala, Sweden; 3 Department of Animal Health and Antimicrobial Strategies, National Veterinary Institute (SVA), SE-75189 Uppsala, Sweden

**Keywords:** Antimicrobial resistance, Extended-spectrum cephalosporinases, Plasmid-mediated quinolone resistance

## Abstract

This study investigated the occurrence of antimicrobial-resistant *Escherichia coli* in dairy calves in southern Vietnam. Fecal samples were taken directly from the rectum of 84 calves from 41 smallholder dairy farms, when newborn and at 14 days of age for isolation of *E. coli. Escherichia coli* strains were isolated from 144 of the 168 fecal samples tested. Of the 144 *E. coli* isolates, 40% were found to be susceptible to all 12 antimicrobial drugs tested and 53% of the *E. coli* isolates were resistant to at least three antimicrobials. Calves were colonized with antimicrobial-resistant *E. coli* already on the day of birth. Resistance to tetracycline was most common, followed by resistance to sulfamethoxazole, ampicillin, trimethoprim, and ciprofloxacin. Four isolates carried a gene encoding for extended-spectrum cephalosporinases (ESC), and these genes belonged to *bla*_CTX-M_ group 1 (2 isolates), *bla*_CTX-M_ group 9 (1 isolate), and *bla*_*CMY-2*_ (1 isolate). Thirty-three isolates had a plasmid-mediated quinolone resistance (PMQR) phenotype, and 30 of these carried the *qnr*S gene. These results are of importance for management routines of dairy cattle to prevent the spread of antimicrobial resistance.

## Introduction

The emergence and spread of antimicrobial-resistant bacteria is an increasing problem and a threat to global public health (WHO 2017). Due to the development of antimicrobial resistance, the European Union banned the use of growth-promoting antimicrobials in animal production in 2006 (European Council 2006). The Ministry of Agriculture and Rural Development (MARD) in Vietnam has had regulations banning some antimicrobials for growth-promoting purposes in animal production since 2002 (MARD 2002).

In Vietnam, 70% of the drug products used in animal production are antimicrobials (An 2009). Antimicrobial consumption has contributed to high levels of antimicrobial-resistant fecal bacteria in calves (Pereira et al. 2014). *Escherichia coli*, a commensal bacterium of the gastrointestinal tract in both animals and humans, are frequently used as an indicator to monitor antimicrobial resistance in fecal samples. The few publications available on the use of antimicrobials and problems with antimicrobial resistance in Southeast Asia (SE Asia) have primarily focused on pig, chicken, fish, and shrimp production (Nhung et al. 2016).

Resistant organisms or their genes can be transmitted from animals to humans by direct contact, via the food chain, or through environmental contamination (Iglesias et al. 2012) and are therefore of great concern for human health. Production of extended-spectrum cephalosporinases (ESC) is one of the most common mechanisms of oxyiminocephalosporin resistance in *E. coli* (Pitout and Laupland 2008). The *bla*_*CTX-M*_ gene has been detected in a large proportion of ESC-producing *E. coli* isolated from dairy calves, chicken meat, and pork (Le et al. 2015; Awosile et al. 2018). The prevalence of antimicrobial-resistant bacteria, especially quinolone-resistant bacteria in chickens, is higher in SE Asia countries (Indonesia, Thailand, and Vietnam) than in developed countries (Usui et al. 2014). Quinolones are critically important antimicrobials for treating severe infections in humans (WHO 2017), and reduced susceptibility to quinolone can lead to treatment failure and is considered a public health risk (Gagliotti et al. 2008). Holloway et al. (2017) reported that antimicrobial drugs are sold without prescription in SE Asia; these countries have high antimicrobial use and poor implementation of polices to promote suitable use. Based on the findings, it can be assumed that quinolone resistance and plasmid-mediated quinolone resistance (PMQR) are more common in dairy calves in SE Asia than in countries with more restrictive antimicrobial use. However, little information is available to date about the prevalence of antimicrobial resistance microbes in dairy calves in SE Asia.

The main aim of this study was therefore to investigate the occurrence of antimicrobial-resistant *E. coli* in samples from young dairy calves on smallholder dairy farms in southern Vietnam, with special emphasis on PMQR and ESC resistance because of their potential transfer to human pathogens.

## Materials and methods

### Farms and sampling

The study was conducted on 41 smallholder dairy farms located in Dong Nai province, southern Vietnam. A total of 84 dairy calves were available for sampling in the study period (August to December), from 5 to 10 dairy cows per farm and with at least two calves born during the period. Each farm was visited twice, with 14 days between the visits. Fecal samples were taken directly from the rectum of calves when newborn and at 14 days of age, and placed in sterile tubes. The tubes were immediately placed on ice and transferred to the laboratory for isolation of *E. coli*. In total, 168 fecal samples were collected.

### Sample analysis

#### *Escherichia coli* culture and isolation

From each sample, fecal material was streaked onto MacConkey agar (Neogen, Michigan, USA) using a sterile cotton-tipped swab and incubated overnight at 37 °C. Five lactose-fermenting (bright pink) colonies with typical *E. coli* morphology were randomly selected from the MacConkey agar plate. These colonies were subcultured on horse blood agar plates (Neogen, Michigan, USA), incubated overnight at 37 °C, and tested for production of tryptophanase (indole) using the spot indole test (Miller and Wright 1982). Lactose- and indole-positive isolates with typical colony morphology (bright pink on MacConkey agar, blue in spot indole test and single-colony types) were considered *E. coli*. Confirmed isolates of *E. coli* were transferred to 2-mL microtubes (SARSTEDT, Nümbrecht, Germany) containing 0.5 mL serum broth supplemented with 15% glycerol. The microtubes were placed in a freezer at − 80 °C. One of the five frozen isolates of *E. coli* from each calf sample was selected at random and sent frozen (on dry ice) to the National Veterinary Institute, Uppsala, Sweden. Directly upon arrival, isolates were transferred to a freezer at − 20 °C and stored until further testing.

#### Antimicrobial susceptibility testing

For each isolate, the minimum inhibitory concentration (MIC) to 12 common antimicrobials was determined using broth microdilution. Testing was performed according to recommendations by the Clinical and Laboratory Institute (CLSI 2013) using VetMIC panels (National Veterinary Institute, Uppsala, Sweden) and cation-adjusted Mueller Hinton Broth (Becton Dickinson, Cockeysville, MD, USA). Epidemiological cut-off values (ECOFFs) set by the European Committee on Antimicrobial Susceptibility Testing were used to classify isolates as susceptible or resistant. Antimicrobials, ranges, and ECOFFs are given in Table 1. Quality control, using the reference strain *E. coli* ATCC 25922, was conducted in parallel with each batch of isolates and all results were within acceptable ranges.

**Table 1:**
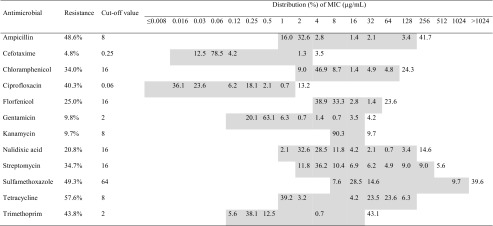
Distribution of the minimum inhibitory concentration (MIC) of 12 common antimicrobials in fecal *E. coli* from newborn calves and calves aged 14 days (84 calves, 144 isolates)^1^

^1^The shaded area represents range of antimicrobial tested

Isolates with cefotaxime MIC > 0.25 μg/mL were phenotypically tested for production of ESC with broth microdilution using EUVSEC2 panels (Trek Diagnostic System, Oakwood Village, OH, USA) and cation-adjusted Mueller Hinton broth (Becton Dickinson, Cockeysville, MD, USA). Isolates with a cefotaxime MIC > 0.25 μg/mL or a ceftazidime MIC > 0.5 μg/mL on the EUVSEC2 panels were further screened by multiplex-polymerase chain reaction (PCR) for detection of the following gene groups: plasmid-mediated AmpC (pAmpC) and *bla*_CTX-M_ (Pérez-Pérez and Hanson 2002). Isolates with ertapenem MIC > 0.06 μg/mL or meropenem MIC > 0.125 μg/mL were further characterized by whole genome sequencing (WGS) to identify transferable genes encoding for carbapenemases.

The DNA used for WGS was extracted from colonies on horse blood agar plates, using an EZ1 DNA tissue kit according to the manufacturer’s protocol (Qiagen, Hilden, Germany). The DNA concentration was determined using Qubit HS DNA-kit (Life Technologies, Carlsbad, CA, USA). Samples were submitted to GATC Biotech (Konstanz, Germany) and subjected to standard genomic library preparation followed by 2 × 150 bp paired-end sequencing using Illumina-based technology. To identify potential contamination and for confirmation of bacterial species, reads were checked using KRAKEN against the pre-built MiniKRAKEN 8GB database (2017-10-19) (Wood and Salzberg 2014). Reads were trimmed with Trimmomatic 0.36 and genome assembly was performed with SPAdes v.3.11.1 with the “- -careful” parameter and an input average coverage of 50×, followed by Pilon v1.22 with default settings to correct assemblies (Bolger et al. 2014). Presence of potential genes encoding antibiotic resistance was checked using Antimicrobial Resistance Identification By Assembly (ARIBA) v2.11.1 (Hunt et al. 2017), together with the downloaded databases of CARD v2.0.1 and ResFinder (2018-06-07) (McArthur and Wright 2015).

Isolates with ciprofloxacin MIC > 0.06 μg/mL and nalidixic acid MIC < 32 μg/mL were selected for PCR detection of plasmid-mediated quinolone resistance (PMQR) genes. The screening for PMQR genes included *qnrA*, *qnrB*, *qnrS*, and *aac(6′)-1b-cr*, using PCR assays described earlier (Cavaco et al. 2008).

### Statistical analysis

Fisher’s exact test was used to compare the observed proportions of resistance to each antimicrobial compound in isolates from samples obtained from calves aged 0 and 14 days. The significance level was set to *p* < 0.05. All statistical analyses were conducted in Stata 13 (StataCorp. 2013. Stata Statistical Software: Release 13. College Station, TX, USA).

## Results

From the total of 168 fecal samples, *E. coli* was isolated in 144 samples and was not detected in 24 samples (21 samples from newborn calves, three from 14-day-old calves). These *E. coli* isolates were subjected to antimicrobial susceptibility testing, the results of which are presented in Table 1. Of the 144 *E. coli* isolates, 40% were found to be pan-susceptible (defined as susceptible to all antimicrobial drugs tested) and 53% were multidrug-resistant (defined as resistant to at least three antimicrobial drugs). Resistance to tetracycline was most common (57.6% of the isolates), followed by sulfamethoxazole (49.3%), ampicillin (48.6%), trimethoprim (43.8%), and ciprofloxacin (40.3%).

Seven isolates had a cefotaxime MIC > 0.25 μg/mL and were eligible for phenotypic ESC screening, and six of these were sent for genotypic screening based on the phenotypic results (Table 2). The phenotypic and genotypic screening confirmed that four isolates could be considered ESC producers. Two of these carried a gene belonging to *bla*_CTX-M_ group 1 (from the same calf on two sampling occasions), another isolate carried a gene belonging to *bla*_CTX-M_ group 9, and the fourth, a gene belonging to the pAmpC-producing gene group *bla*_*CMY*_ group 2. The two remaining isolates of the six screened also exceeded the ECOFFs for ertapenem and meropenem, and thus showed carbapenem resistance. However, no transferrable genes that could explain the carbapenem resistance phenotype were identified. The seventh isolate was below the cefotaxime and ceftazidime ECOFFs, and was thus not of interest for further screening.

**Table 2:** Results of phenotypic and genotypic screening for isolates producing extended-spectrum cephalosporinases (ESC) for seven isolates with cefotaxime MIC > 0.25 μg/mL obtained from feces from dairy calves. ECOFF, epidemiological cut-off value

	Antimicrobial										ESC gene group
	Cefepime	Cefotaxime	Cefotaxime and clavulanic acid	Cefoxitin	Ceftazidime	Ceftazidime and clavulanic acid	Ertapenem	Imipenem	Meropenem	Temocillin	
Range tested	0.06–32	0.25–64	0.06/4–64/4	0.5–64	0.25–128	0.12/4–128/4	0.015–2	0.12–16	0.03–16	4–128	
ECOFF	0.125	0.25	NA	8	0.5	NA	0.064	0.5	0.125	> 32	
ID no.											
E 11.1.1	4	64	≤ 0.06	2	4	≤ 0.12	≤ 0.015	≤ 0.12	≤ 0.03	4	*bla*CTX-M-1
E 11.1.2	4	32	≤ 0.06	4	4	≤ 0.12	≤ 0.015	≤ 0.12	≤ 0.03	4	*bla*CTX-M-1
E 14.2.2	≤ 0.06	4	1	8	4	2	≤ 0.015	≤ 0.12	≤ 0.03	2	*CIT*
E 21.2.1	32	> 64	> 64	> 64	> 128	> 128	> 2	1	4	> 128	No genes found
E 33.2.1	1	8	8	> 64	2	1	2	1	1	> 128	No genes found
E 42.2.1	≤ 0.06	≤ 0.25	≤ 0.06	2	≤ 0.25	≤ 0.12	≤ 0.015	≤ 0.12	≤ 0.03	4	ND
E 57.2.2	4	64	≤ 0.06	2	0.5	≤ 0.12	≤ 0.015	≤ 0.12	≤ 0.03	4	*bla*CTX-M-9

*NA*, not applicable; *ND*, not done

Thirty-three isolates were eligible for PMQR screening, based on their resistance profile of ciprofloxacin and nalidixic acid, of which 26 were from samples collected at 14 days of age. Thirty of the screened isolates (21% of all isolates) carried the *qnrS* gene, but none of the other genes, i.e., *qnrA*, *qnrB*, and *aac(6′)-1b-cr*, was detected. The 30 PMQR-positive strains were from 28 calves located on 22 different farms.

**Fig. 1:**
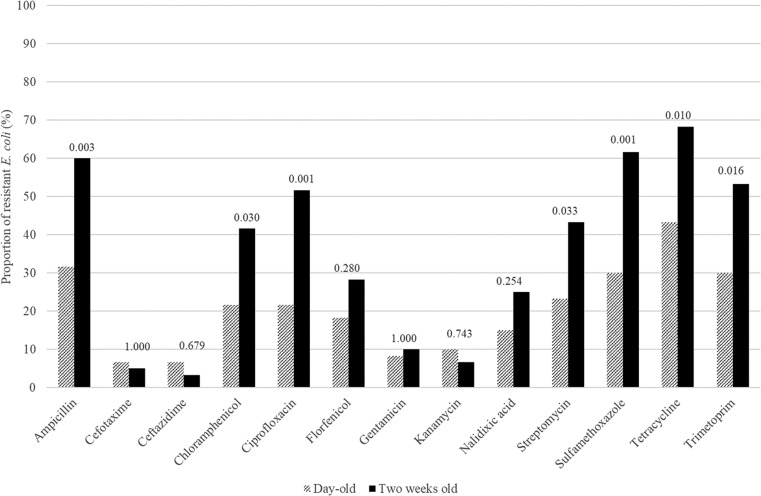
Proportion of antimicrobial-resistant *Escherichia coli* sampled from feces from the same group of 60 calves on two occasions, when newborn and at 14 days old. *P* values indicate the results of Fisher’s exact test on differences in the proportion of antimicrobial-resistant isolates at the two sampling occasions (*P* < 0.05 indicates significantly different proportions)

The proportions of antimicrobial-resistant *E. coli* in samples from the same calves when newborn and at 14 days of age are shown in Fig. 1. Significantly (*P* < 0.05), larger proportions of isolates from samples taken at 14 days of age were resistant to ampicillin, chloramphenicol, ciprofloxacin, streptomycin, sulfamethoxazole, tetracycline, and trimethoprim. No significant difference was observed for the remaining antimicrobials tested.

## Discussion

The incidence of antimicrobial resistance (around 60%) among the *E. coli* isolates in the present study was higher than reported previously in Sweden (Duse et al. 2015a). This is most likely the result of high use of antimicrobials such as beta-lactams, aminoglycosides, fluoroquinolones, and macrolides in Vietnam (Carrique-Mas et al. 2015).

The incidence of resistance to ampicillin, tetracycline, and sulfamethoxazole was 48.6–57.6% among the 144 isolates tested. These antimicrobial resistance types have been found in over 50% of *E. coli* isolates from pigs and chickens in SE Asia (Nhung et al. 2016). The high proportion of tetracycline and ampicillin resistance probably reflects the long history of using these antimicrobials for treatment and prophylactic purposes (Tadesse et al. 2012). Chloramphenicol is banned for use in animal feed (MARD 2002) in Vietnam. However, chloramphenicol resistance was still high (34.0% of the isolates) in the present study, which might be due to chloramphenicol still being used in animal production (Nhung et al. 2016). Moreover, thiamphenicol and florfenicol, which belong to the same family as chloramphenicol, are still permitted for use in livestock and aquaculture (MARD 2010) and chloramphenicol resistance is commonly co-selected with these antimicrobials (Harada et al. 2006).

The high proportion of multidrug-resistant strains found in the present study might be a result of the common use of combinations of antimicrobials and of broad-spectrum antimicrobials in this region, as discussed in a previous study (Ström et al. 2017). It could also be a case of co-selection (Harada et al. 2006). It was previously documented that resistant strains selected during an antimicrobial treatment last for a long time in the intestinal tract when this treatment ceases. Additionally, these resistant strains could modify animal health and can be transmitted to other animals, especially to their offspring and accompanying animals (Roca-Saavedra et al. 2018).

Antimicrobial-resistant strains of *E. coli* were observed in newborn calves in the present study. Antimicrobials and resistant organisms are spread to the environment through wastes (feces, slurry, and wastewater) from agricultural animals (da Costa et al. 2013). A high level of general antimicrobial use might lead to a high proportion of resistant strains in the environment, and thus the *E. coli* that first enters the gastrointestinal tract of the calf is highly likely to be antimicrobial-resistant.

The proportions of ampicillin, chloramphenicol, ciprofloxacin, streptomycin, sulfamethoxazole, tetracycline, and trimethoprim resistance were significantly lower in newborn calves than in the same calves at 14 days of age. Earlier studies have found that resistant strains dramatically colonize dairy calves after parturition, but that the prevalence of antimicrobial-resistant strains commonly peaks in calves at 14 days of age (Donaldson et al. 2006) and thereafter declines in calves aged 4–6 weeks (Berge et al. 2005). The reason is a transition from susceptible to resistant strains, and then back to susceptible strains, in the gut of the developing calf, and there is no reemergence of susceptible strains that have been dominant when the calves are younger (Hinton et al. 1985).

In the present study, ESC-producing *E. coli* was found in about 3% of the 144 isolates. However, only non-selective methods were used to detect ESC-producing isolates, so the true prevalence of ESC-producing *E. coli* is probably much higher. The ESC-producing gene groups (*bla*_CTX-M_ and *bla*_*CMY*_) found in the present study have been observed previously in Holstein dairy calves in Canada (Awosile et al. 2018). Moreover, *bla*_CTX-M-1_, and *bla*_CTX-M-9_ are the main β-lactamase resistance groups detected in *E. coli* isolates from chicken meat and pork in Vietnam (Le et al. 2015). Although two isolates showed phenotypic resistance to carbapenem, it was not possible to confirm this by genotypic characterization of these isolates after WGS. The reason could be that the gene encoding for carbapenem resistance was lost during repeated freezing and thawing cycles of the stored isolate, or that phenotypic resistance is encoded by genes not yet described. It could also be the case of a combination of chromosomal AmpC and porin loss.

The incidence of quinolone-resistant *E. coli* (QREC) isolates was 21%, although fluoroquinolone drugs are not used on the farms tested. One explanation could be the horizontal spread of PMQR genes from the surroundings, as the dairy farmers apply irrigation water, livestock wastewater, and manure to grass crops, possibly increasing the abundance of fluoroquinolones and antimicrobial resistance genes in the environment, as documented in recent studies (McKinney et al. 2018). There might also be some environmental cross-contamination of fluoroquinolones from human sanitary systems (Phu et al. 2016). The incidence of QREC was higher than reported previously in pre-weaned dairy calves (Duse et al. 2015b). A large proportion (91%) of the isolates eligible for PMQR screening showed the presence of PMQR genes, and to our knowledge, this is a unique finding in dairy calves in Vietnam. The *qnrS* gene has also been found in *E. coli* isolates from dairy calves in Canada (Awosile et al. 2018). Humans live in close proximity to calves in Vietnam, and thus, there is a high risk of these genes spreading to human pathogens, such as *Salmonella* (Veldman et al. 2011).

To our knowledge, this is the first report of antimicrobial resistance in dairy cattle and occurrence of ESC and *qnrS* genes in *E. coli* isolated from dairy calves in Vietnam. These findings should be taken into consideration by veterinarians/policy makers and by dairy farmers when devising management regimes for dairy cattle in order to combat antimicrobial resistance. They can also be used as a starting point for future antimicrobial resistance monitoring in Vietnam.

## References

[CR1] An, N.Q., 2009. Report of antibiotic use in animals in Viet Nam. Hanoi: Global Antibiotics Resistance Partnership (GARP) Workshop.

[CR2] Awosile B, McClure J, Sanchez J, Rodriguez-Lecompte JC, Keefe G, Heider LC (2018). *Salmonella enterica* and extended-spectrum cephalosporin-resistant *Escherichia coli* recovered from Holstein dairy calves from 8 farms in New Brunswick, Canada. Journal of Dairy Science.

[CR3] Berge A, Atwill E, Sischo W (2005). Animal and farm influences on the dynamics of antibiotic resistance in faecal *Escherichia coli* in young dairy calves. Preventive Veterinary Medicine.

[CR4] Bolger AM, Lohse M, Usadel B (2014). Trimmomatic: a flexible trimmer for Illumina sequence data. Bioinformatics.

[CR5] Carrique-Mas JJ, Trung NV, Hoa NT, Mai HH, Thanh TH, Campbell JI, Wagenaar JA, Hardon A, Hieu TQ, Schultsz C (2015). Antimicrobial usage in chicken production in the Mekong Delta of Vietnam. Zoonoses and Public Health.

[CR6] Cavaco LM, Frimodt-Møller N, Hasman H, Guardabassi L, Nielsen L, Aarestrup FM (2008). Prevalence of quinolone resistance mechanisms and associations to minimum inhibitory concentrations in quinolone-resistant *Escherichia coli* isolated from humans and swine in Denmark. Microbial Drug Resistance.

[CR7] CLSI, 2013. Performance Standards for Antimicrobial Disk and Dilution Susceptibility Tests for Bacteria Isolated from Animals; Approved Standard (VET01–A4). 4th ed. Clinical and Laboratory Standards Institute (CLSI), Wayne, PA.

[CR8] da Costa PM, Loureiro L, Matos AJ (2013). Transfer of multidrug-resistant bacteria between intermingled ecological niches: the interface between humans, animals and the environment. International Journal of Environmental Research and Public Health.

[CR9] Donaldson SC, Straley BA, Hegde NV, Sawant AA, DebRoy C, Jayarao BM (2006). Molecular epidemiology of ceftiofur-resistant *Escherichia coli* isolates from dairy calves. Applied and Environmental Microbiology.

[CR10] Duse A, Waller KP, Emanuelson U, Unnerstad HE, Persson Y, Bengtsson B (2015). Risk factors for antimicrobial resistance in fecal *Escherichia coli* from preweaned dairy calves. Journal of Dairy Science.

[CR11] Duse A, Waller KP, Emanuelson U, Unnerstad HE, Persson Y, Bengtsson B (2015). Risk factors for quinolone-resistant *Escherichia coli* in feces from preweaned dairy calves and postpartum dairy cows. Journal of Dairy Science.

[CR12] European Council, 2006. Regulation (EC) No. 1831/2003 of the European Parliament and of the Council of 22 September 2003 on additives for use in animal nutrition.

[CR13] Gagliotti C, Buttazzi R, Sforza S, Moro ML, Group, E.-R.A.R.S (2008). Resistance to fluoroquinolones and treatment failure/short-term relapse of community-acquired urinary tract infections caused by *Escherichia coli*. Journal of Infection.

[CR14] Harada K, Asai T, Kojima A, Ishihara K, Takahashi T (2006). Role of coresistance in the development of resistance to chloramphenicol in *Escherichia coli* isolated from sick cattle and pigs. American Journal of Veterinary Research.

[CR15] Hinton M, Linton A, Hedges A (1985). The ecology of *Escherichia coli* in calves reared as dairy-cow replacements. Journal of Applied Microbiology.

[CR16] Holloway KA, Kotwani A, Batmanabane G, Puri M, Tisocki K (2017). Antibiotic use in South East Asia and policies to promote appropriate use: reports from country situational analyses. BMJ.

[CR17] Hunt, M., Mather, A.E., Sánchez-Busó, L., Page, A.J., Parkhill, J., Keane, J.A. and Harris, S.R., 2017. ARIBA: rapid antimicrobial resistance genotyping directly from sequencing reads. Microbial Genomics, 3. 10.1099/mgen.0.000131 PMC5695208 29177089

[CR18] Iglesias A, Nebot C, Miranda JM, Vázquez BI, Cepeda A (2012). Detection and quantitative analysis of 21 veterinary drugs in river water using high-pressure liquid chromatography coupled to tandem mass spectrometry. Environmental Science and Pollution Research.

[CR19] Le QP, Ueda S, Nguyen TNH, Dao TVK, Van Hoang TA, Tran TTN, Hirai I, Nakayama T, Kawahara R, Do TH (2015). Characteristics of Extended-Spectrum β-Lactamase–Producing *Escherichia coli* in Retail Meats and Shrimp at a Local Market in Vietnam. Foodborne Pathogens and Disease.

[CR20] MARD, 2002. Desision No. 54/2002/QĐ-BNN dated June 20th, 2002 of Ministry of Agriculture and Rural Development on Banned production, impertation, circulation and use of some antibiotics and chemicals in the manufacturing and trading of livestock feed.

[CR21] MARD, 2010. Circular No. 69/2010/TT-BNNPTNT dated December 3, 2010 of Ministry of Agriculture and Rural Development on the list of veterinary drugs, bio-preparations, microorganisms and chemicals for use in aquatic animal medicine permitted for circulation in Vietnam.

[CR22] McArthur AG, Wright GD (2015). Bioinformatics of antimicrobial resistance in the age of molecular epidemiology. Current Opinion in Microbiology.

[CR23] McKinney, C.W., Dungan, R.S., Moore, A. and Leytem, A.B., 2018. Occurrence and abundance of antibiotic resistance genes in agricultural soil receiving dairy manure. FEMS Microbiology Ecology. 10.1093/femsec/fiy010 29360961

[CR24] Miller JM, Wright JW (1982). Spot indole test: evaluation of four reagents. Journal of Clinical Microbiology.

[CR25] Nhung NT, Cuong NV, Thwaites G, Carrique-Mas J (2016). Antimicrobial usage and antimicrobial resistance in animal production in Southeast Asia: a review. Antibiotics.

[CR26] Pereira R, Siler J, Ng J, Davis M, Grohn Y, Warnick L (2014). Effect of on-farm use of antimicrobial drugs on resistance in fecal *Escherichia coli* of preweaned dairy calves. Journal of Dairy Science.

[CR27] Pérez-Pérez FJ, Hanson ND (2002). Detection of plasmidmediated AmpC beta-lactamase genes in clinical isolates by usingmultiplex PCR. Journal of Clinical Microbiology.

[CR28] Phu VD, Wertheim HF, Larsson M, Nadjm B, Dinh Q-D, Nilsson LE, Rydell U, Le TTD, Trinh SH, Pham HM (2016). Burden of hospital acquired infections and antimicrobial use in Vietnamese adult intensive care units. PLoS One.

[CR29] Pitout JD, Laupland KB (2008). Extended-spectrum β-lactamase-producing Enterobacteriaceae: an emerging public-health concern. The Lancet Infectious Diseases.

[CR30] Roca-Saavedra P, Mendez-Vilabrille V, Miranda JM, Nebot C, Cardelle-Cobas A, Franco CM, Cepeda A (2018). Food additives, contaminants and other minor components: effects on human gut microbiota—a review. Journal of Physiology and Biochemistry.

[CR31] Ström G, Halje M, Karlsson D, Jiwakanon J, Pringle M, Fernström L-L, Magnusson U (2017). Antimicrobial use and antimicrobial susceptibility in *Escherichia coli* on small-and medium-scale pig farms in north-eastern Thailand. Antimicrobial Resistance and Infection Control.

[CR32] Tadesse DA, Zhao S, Tong E, Ayers S, Singh A, Bartholomew MJ, McDermott PF (2012). Antimicrobial drug resistance in *Escherichia coli* from humans and food animals, United States, 1950–2002. Emerging Infectious Diseases.

[CR33] Usui M, Ozawa S, Onozato H, Kuge R, Obata Y, Uemae T, Ngoc PT, Heriyanto A, Chalemchaikit T, Makita K (2014). Antimicrobial susceptibility of indicator bacteria isolated from chickens in Southeast Asian countries (Vietnam, Indonesia and Thailand). Journal of Veterinary Medical Science.

[CR34] Veldman K, Cavaco LM, Mevius D, Battisti A, Franco A, Botteldoorn N, Bruneau M, Perrin-Guyomard A, Cerny T, De Frutos Escobar C (2011). International collaborative study on the occurrence of plasmid-mediated quinolone resistance in *Salmonella enterica* and *Escherichia coli* isolated from animals, humans, food and the environment in 13 European countries. Journal of Antimicrobial Chemotherapy.

[CR35] WHO, 2017. World Health Organization. Critically important antimicrobials for human medicine: ranking of antimicrobial agents for risk management of antimicrobial resistance due to non-human use.

[CR36] Wood DE, Salzberg SL (2014). Kraken: ultrafast metagenomic sequence classification using exact alignments. Genome Biology.

